# Metformin treatment and risk of diabetic peripheral neuropathy in patients with type 2 diabetes mellitus in Beijing, China

**DOI:** 10.3389/fendo.2023.1082720

**Published:** 2023-02-28

**Authors:** Ruotong Yang, Huan Yu, Junhui Wu, Hongbo Chen, Mengying Wang, Siyue Wang, Xueying Qin, Tao Wu, Yiqun Wu, Yonghua Hu

**Affiliations:** ^1^ Department of Epidemiology and Biostatistics, School of Public Health, Peking University Health Science Center, Beijing, China; ^2^ School of Nursing, Peking University, China, Beijing, China; ^3^ Medical Informatics Center, Peking University, Beijing, China

**Keywords:** metformin, diabetic peripheral neuropathy, type 2 diabetes, Beijing Medical Claim Data for Employees database, cohort study

## Abstract

**Background:**

Metformin treatment is associated with vitamin B12 deficiency, which is a risk factor for neuropathy. However, few studies have examined the relationship between metformin treatment and diabetic peripheral neuropathy (DPN), and the available findings are contradictory. We aimed to assess whether metformin treatment is associated with DPN in patients with type 2 diabetes mellitus (T2DM) in Beijing, China.

**Methods:**

All patients with newly diagnosed T2DM between January 2010 and September 2012 in the Medical Claim Data for Employees database were included. Metformin treatment was defined as any record of metformin prescription. The average daily dose of metformin during follow-up was calculated. DPN was defined as DPN admissions occurring after a diagnosis of T2DM in the database. Hazard ratios (HRs) and 95% confidence intervals (CIs) were calculated using Cox proportional hazards models.

**Results:**

Among 49,705 T2DM patients, 1,933 DPN events were recorded during a median follow-up of 6.36 years. The crude incidence rates were 7.12 and 3.91 per 1000 person-years for patients treated with metformin (N=37,052) versus those not treated (N=12,653). Patients treated with metformin had an 84% increased risk of DPN compared with patients not using metformin (HR, 1.84; 95% CI, 1.62, 2.10). The daily dose was positively associated with DPN risk (HR, 1.48; 95% CI, 1.46, 1.51; P for trend <0.001). The risk of DPN was 1.53-fold (1.30, 1.81) and 4.31-fold (3.76, 4.94) higher in patients with daily doses of 1.0-2.0 g and >2.0 g, respectively, than in patients who did not receive treatment. Patients aged less than 60 years had a higher risk of DPN (P<0.05 for interaction test). Among patients taking vitamin B12 at baseline, there was no increased risk of DPN in the metformin group (1.92: 0.79, 4.69).

**Conclusions:**

In Chinese patients with T2DM, metformin treatment was associated with an increased risk of DPN admission and this risk responds positively to the daily dose of metformin. In particular, metformin use was a major risk factor for DPN in younger patients. Concomitant use of vitamin B12 may avoid the increased risk of DPN associated with metformin use.

## Introduction

Diabetic peripheral neuropathy (DPN) is the most prevalent chronic complication of diabetes, affecting 30–50% of patients with diabetes (1). DPN is a leading cause of diabetic foot, lower-limb amputations and disabling neuralgia, with devastating effects on quality of life and a significant reduction in life expectancy. Although approximately half of patients with DPN may be asymptomatic, once DPN develops, it is extremely difficult to treat and incurs a range of additional medical costs (2).

Metformin is one of the most widely used oral glucose-lowering drugs for the treatment of type 2 diabetes and has been prescribed to more than 100 million people worldwide (3). However, metformin treatment has been reported to be associated with vitamin B12 deficiency, potentially leading to irreversible nervous system damage and an increased risk of DPN (4, 5). To date, direct evidence of the association between metformin treatment and DPN risk is scarce and conflicting (6). Several cross-sectional studies with small sample sizes (<500 patients) found no significant association between metformin use and the prevalence of DPN or neuropathy scores (7–10). A retrospective cohort study involving 210,004 elderly veterans found that long-term metformin use was associated with an elevated risk of DPN (11), and another case−control study involving 150 patients found that metformin use for the previous 6 months was associated with an increased risk of moderate-to-severe DPN, particularly in the high-dose group (12).

Therefore, the aim of this study was to assess the association between metformin treatment and the risk of DPN in Chinese patients with type 2 diabetes mellitus (T2DM) to complement the evidence from Asian populations.

## Methods

### Data source

This study was based on the Beijing Medical Claim Data for Employees (BMCDE) database, which has been previously described elsewhere (13, 14). Briefly, the database contains anonymized medical claims data (demographic characteristics, clinical diagnosis, medications and reimbursement information) for all active or retired employees enrolled in basic medical insurance in Beijing. By the end of 2017, nearly 90% of Beijing’s resident population had been included in the database. Clinical diagnosis information was presented in the International Classification of Diseases, 10th Revision (ICD-10) codes as well as descriptive texts. Drug information includes brand and generic drug names, formulations, costs, and dispensing dates. Our research was exempt from ethics committee review because of the use of encrypted retrospective information for administrative purposes.

### Study population

**Figure 1:**
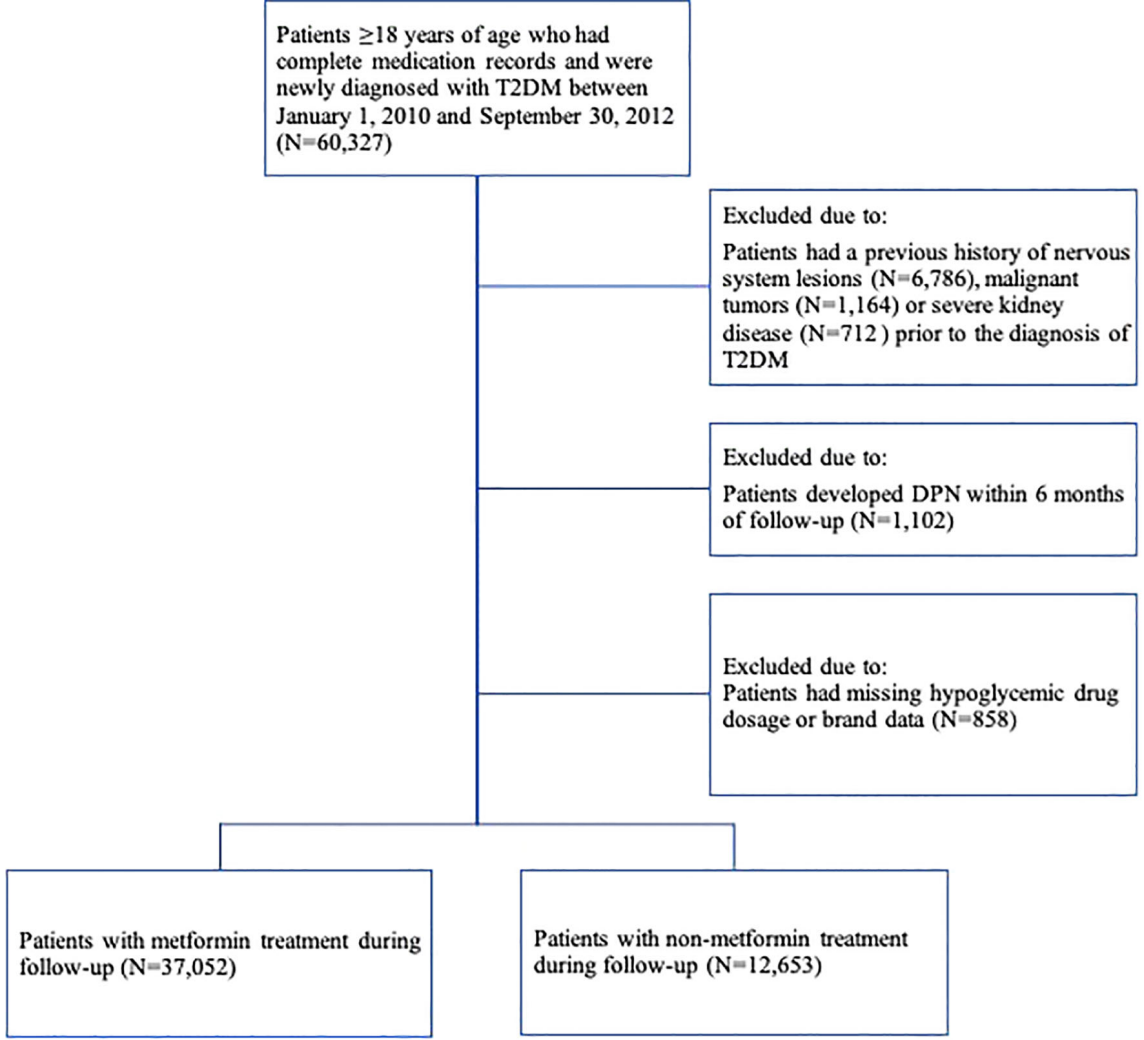
Subject Selection Criteria.

The study population was selected from patients ≥18 years of age who were newly diagnosed with T2DM between January 1, 2010, and September 30, 2012 (N=60,327). Patients with T2DM were identified by ICD-10 coding (E11-E14) and text diagnosis. “Newly diagnosed” was defined by applying a fixed 24-month look-back period in which the patient had continuous data coverage but no ICD-10 records (E11-E14) or textual diagnosis of diabetes. If a patient had multiple records of dispensation episodes, only the first one was used. The date of first diagnosis of T2DM was determined as the baseline index date. Subjects were excluded if they (**Figure 1**) (1) had a previous history of primary diagnosis of nervous system lesions (ICD-10: G00-G99 and text diagnosis), malignant tumors (ICD-10: C00-C75, C76-C80 and text diagnosis) or severe kidney disease (ICD-10: I12; I13; N00-N05; N07; N11; N14; N17-N19; Q61 and text diagnosis) before the date of diagnosis of T2DM (15) (2), developed DPN within 6 months of follow-up, or (3) lacked data on the dosage or brand of antidiabetic drugs.

### Metformin exposure

Medication use information included the trade name, dosage, and prescription date of all prescribed drugs for all patients. Metformin treatment in this study was defined as any metformin prescription recorded after diagnosis of T2DM from 2010 through 2017 (N=37,052). Non-metformin treatment was considered as no metformin prescription recorded during follow-up (N=12,653). The average daily dose of metformin was equal to the total dose divided by the total number of days of follow-up. According to the daily dose of metformin, patients were divided into four groups: non-metformin, metformin <1.0 g/d, 1.0-2.0 g/d and >2.0 g/d.

### Ascertainment of DPN

DPN was defined as receiving any of the ICD-10 diagnosis codes (G63.2) or text diagnosis during the study period. As previously described, we excluded those who developed DPN within 6 months to avoid baseline disease risk. Patients were followed from the date of diagnosis of T2DM (baseline) to the earliest outcome occurrence, death, withdrawal from the database, or study termination on December 31, 2017, whichever came first.

### Covariates

Baseline demographic characteristics included sex and age at baseline. Comorbidity history was determined by ICD-10 codes and text diagnosis from inpatient and outpatient case records from January 1, 2008, to baseline date. The Charlson comorbidity index was established from comorbidity history to reflect the baseline health status of the study population, as described in a previous study (15). Medical resource utilization was measured by the number of hospital visits in the 12 months prior to baseline. In addition, baseline concomitant prescription medication records were included in this study, including antihypertensive, antihyperlipidemic, non-steroidal anti-inflammatory drugs (NSAIDs), neurotrophic drugs (vitamins B1 and B12) and other types of hypoglycemic drugs.

### Statistical analysis

Baseline characteristics, Charlson comorbidity, medical resource utilization, and concomitant medication use were expressed as the means (and standard deviations) of continuous variables and the numbers (and percentages) of categorical measures for patients in the metformin-treated and non-treated groups. Unadjusted Kaplan−Meier incidence curves and log-rank tests were used to assess the incidence of DPN in the treatment and non-treatment groups. Cox proportional hazards models were used to estimate the hazard ratios (HRs) and 95% confidence intervals (CIs) for the association between metformin treatment and DPN occurrence, adjusted for sex, age at baseline, comorbidity index, number of visits and concomitant prescription medications. The proportionality risk hypothesis was tested using Schoenfeld residuals (no deviations from proportionality were observed). The risk of DPN was calculated for different daily dose groups, and a dose−response relationship between daily dose and DPN risk was calculated. Because of the varying timing of the first use of metformin, we also examined the DPN risk among people who started metformin at different times after the diagnosis of T2DM compared with those who did not use metformin. Furthermore, we performed stratified analyses by sex, age, comorbidity index, number of visits, and concomitant medications to assess the association of metformin use with the risk of DPN in several subgroups and the interaction of stratification factors with metformin use.

Several additional sensitivity analyses were constructed for robustness of the results by (1) excluding patients without any type of prescription record for glucose-lowering drugs within 6 months to avoid including participants with mild diabetes or inaccurate diagnosis in the non-metformin group at baseline (2); excluding patients who had used insulin within 6 months to avoid including patients with poor glycemic control at the beginning of follow-up (3); excluding patients who had used two or more types of hypoglycemic agents to avoid inclusion of patients with poor glycemic control and to further calculate the relative risk of different hypoglycemic drug treatments (glycosidase inhibitors, metformin, thiazolidinediones, glinides, sulfonylureas or insulin); and (4) matching metformin-treated and non-metformin-treated populations 1:1 within a caliper of 0.01 times the standard error of the logit propensity score (for sex, age, date of diagnosis of T2DM, comorbidity index, number of visits, concomitant medication, and other hypoglycemic agents), and standardized mean difference (SMD) of acceptable matching was less than 0.1 (16). Therefore, the demographic characteristics of the exposed and non-exposed groups were consistent as much as possible (5). Patients who developed DPN within 12 months were excluded to observe the long-term effects of metformin.

All statistical analyses were performed by using Stata (version 14.0, StataCorp). A 2-sided P value of <0.05 was considered to be statistically significant.

## Results

### Baseline characteristics

A total of 37,052 patients with T2DM were treated with metformin, and 12,653 patients were not treated with metformin (**Figure 1**). The distribution of basic characteristics of the two groups was shown in **Table 1**. Specifically, patients in the metformin-treated group were younger, more female, had fewer comorbidities, had lower utilization of health care resources, used anti-hyperlipidemic drugs more frequently, and used vitamin B1, antihypertensive drugs, and NSAIDs less frequently (P <0.05). The high-dose metformin-treated group (>2.0 g/d) had younger patients, a higher proportion of women, more comorbidities, higher health care resource utilization, and more combined drug use (P < 0.05).

**Table 1:** Baseline demographic and clinical characteristics of study participants, divided according to metformin use.

Variable	Non-metformin (N=12,653)	Metformin (N=37,052)	P-value	Daily dose, g			P trend
				<1.0 (N=19,651)	1.0-2.0 (N=8,188)	>2.0 (N=9,213)	
Age, y	64.02 (13.57)	58.17 (12.59)	<0.001	58.38 (13.14)	57.20 (11.92)	58.60 (11.02)	<0.001
Female, %	4,968 (39.26)	16,090 (43.43)	<0.001	7,953 (40.47)	3,603 (44.00)	4,534 (49.21)	<0.001
Comorbidity index	0.95 (0.94)	0.90 (0.85)	<0.001	0.88 (0.85)	0.89 (0.85)	0.93 (0.87)	<0.001
Number of visits/y	15.03 (25.73)	13.96 (25.28)	0.015	13.08 (24.75)	13.64 (24.95)	16.13 (26.51)	<0.001
Medication use, %							
Vitamin B12	349 (2.76)	905 (2.44)	0.051	461 (2.35)	176 (2.15)	268 (2.91)	0.001
Vitamin B1	4,480 (35.41)	12,436 (33.56)	<0.001	6,467 (32.91)	2,576 (31.46)	3,393 (36.83)	<0.001
Antihypertensive	9,733 (76.92)	26,747 (72.19)	<0.001	13,609 (69.25)	5,817 (71.04)	7,321 (79.46)	<0.001
Antihyperlipidemic	7,757 (61.31)	25,271 (68.20)	<0.001	12,742 (64.84)	5,678 (69.35)	6,851 (74.36)	<0.001
NSAIDs	5,924 (46.82)	16,562 (44.70)	<0.001	8,315 (42.31)	3,563 (43.51)	4,684 (50.84)	<0.001

### Metformin therapy and the risk of diabetic peripheral neuropathy

**Figure 2:**
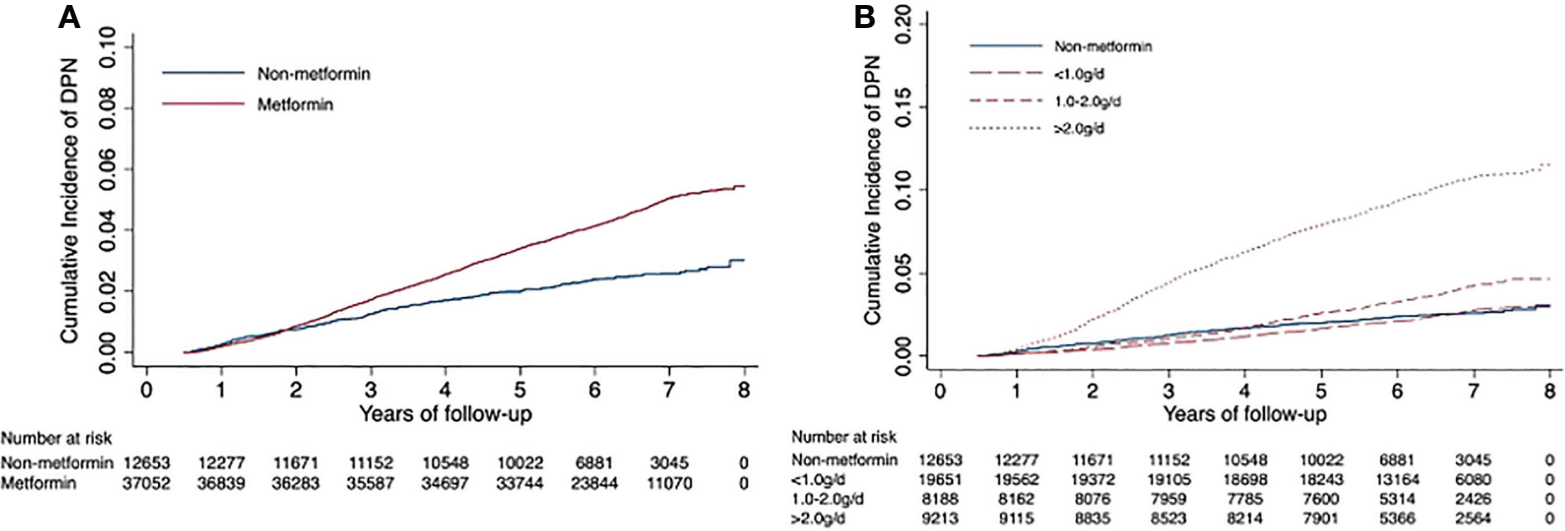
Unadjusted Kaplan−Meier hazard curves for risk of diabetic peripheral neuropathy. Figure **(A)** shows the curves of diabetic peripheral neuropathy for the metformin and non-metformin groups; Figure **(B)** shows the incidence curves for average daily dose groups.

**Figure 3:**
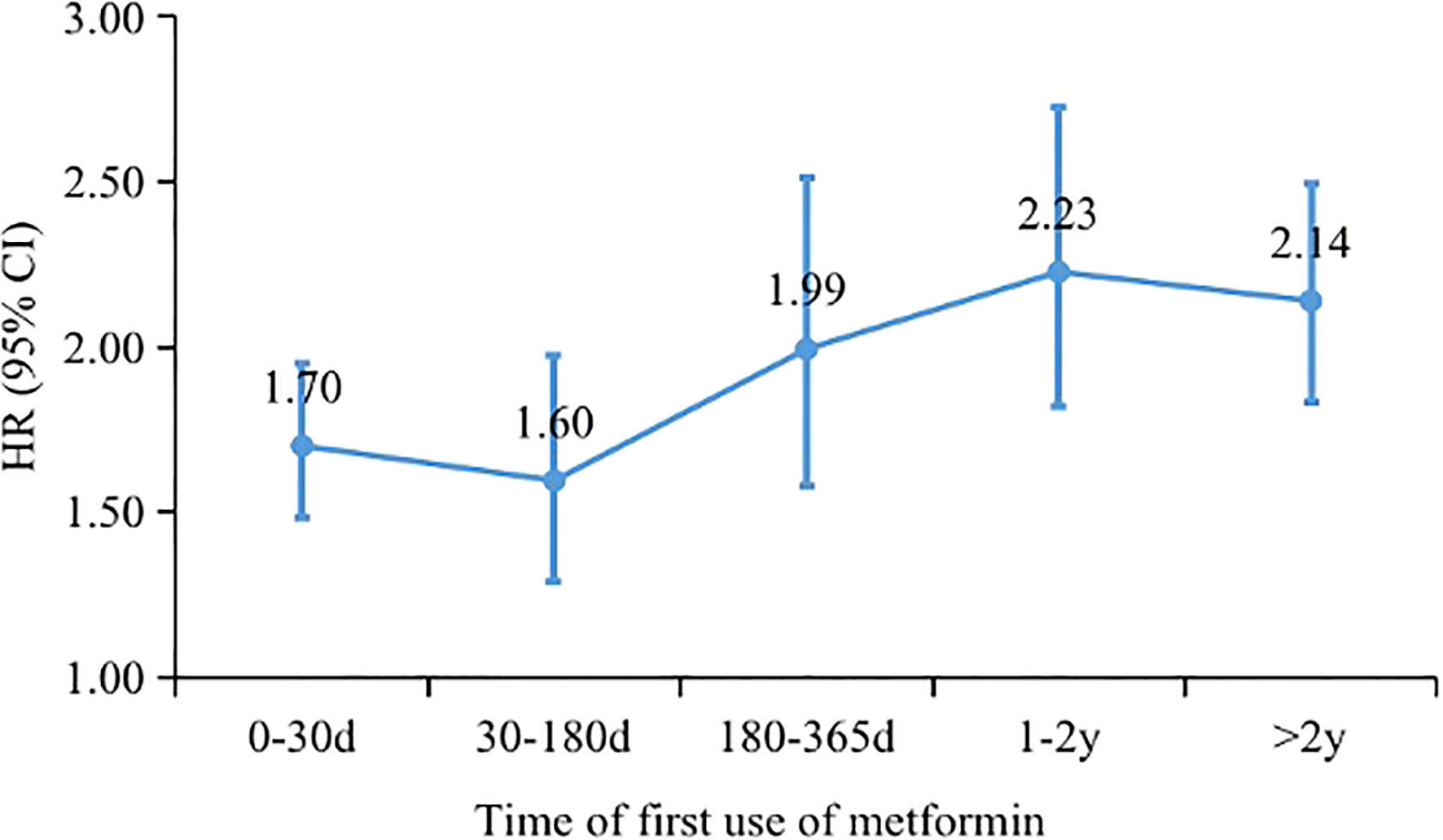
Hazard ratios of diabetic peripheral neuropathy associated with metformin use at different time of metformin initiation after T2DM.

Of the remaining 49,705 patients with T2DM, 1,933 incident cases of DPN were recorded during a median follow-up of 6.36 years. The crude incidence rates were 7.12 and 3.91 per 1000 person-years for patients treated with metformin versus those not treated. In general, patients receiving metformin treatment had a higher risk of DPN than those not receiving metformin treatment, with an adjusted HR of 1.84 (95% CI, 1.62, 2.10). The average daily dose was positively associated with DPN risk (HR, 1.48; 95% CI, 1.46, 1.51; P for trend <0.001). There was no difference in DPN risk in the low-dose (<1.0 g/d) metformin group compared with non-metformin group (0.97: 0.84, 1.13). Patients with daily doses of 1.0-2.0 g and >2.0 g had a 1.53-fold (1.30, 1.81) and 4.31-fold (3.76, 4.94) higher risk of DPN compared with patients not receiving metformin, respectively (**Table 2**). In addition, Kaplan−Meier incidence curves showed that metformin-treated patients had a higher long-term (>2 years of follow-up) risk of DPN than non-metformin-treated patients, with the highest risk in the high-dose group (>2.0 g/d) (P <0.001 for all log-rank tests: **Figure 2**). Throughout the follow-up period from the diagnosis of type 2 diabetes, patients treated had higher risk of DPN compared with those not treated, regardless of when metformin therapy was initiated (**Figure 3**).

**Table 2:** Hazard ratios of diabetic peripheral neuropathy associated with metformin use and average daily dose of metformin use.

	Total	Case	Cases/PYs (/1000)	Crude Hazard Ratio (95% CI)	Adjusted Hazard Ratio* (95% CI)
Non-metformin	12,653	282	3.91	1.00	1.00
Metformin	37,052	1,651	7.12	1.80 (1.59, 2.04)	1.84 (1.62, 2.10)
Daily dose, g	(Continuous)			1.47 (1.44, 1.49)	1.48 (1.46, 1.51)
<1.0	19,651	467	3.75	0.95 (0.82, 1.10)	0.97 (0.84, 1.13)
1.0-2.0	8,188	298	5.77	1.46 (1.24, 1.71)	1.53 (1.30, 1.81)
>2.0	9,213	886	15.97	4.06 (3.55, 4.64)	4.31 (3.76, 4.94)

PY, person year; CI, confidence interval.

*Adjusted for sex, age at baseline, comorbidity index, number of visits and concomitant medication (vitamin B1 and B12, antihypertensive, antihyperlipidemic, and NSAIDs).

In subgroup analyses, we found that patients who were younger than 60 years old (2.29: 1.85, 2.84) or had not seen a doctor a year ago (2.39: 1.92, 2.98) were at higher DPN risk (P <0.05 for all interaction tests: **Table 3**). Metformin treatment did not increase the risk of DPN in patients with vitamin B12 prescription records at baseline (1.92: 0.79, 4.69). In sensitivity analyses, further excluding patients who did not use any hypoglycemic drugs within 6 months, or used insulin within 6 months did not substantially alter the risk estimates (**Supplementary Tables 1, 2**). A higher hazard ratio (2.00: 1.74, 2.29) was obtained by excluding people who developed DPN within 12 months of diabetes diagnosis (**Supplementary Tables 3**). Among patients using only one type of hypoglycemic drug, metformin treatment was associated with a 1.62-fold (1.10, 2.39) higher risk than non-metformin treatment. Compared to treatment with glycosidase inhibitors alone, none of the other types of hypoglycemic agents (thiazolidinediones, glinides, sulfonylureas or insulin) except metformin (2.27: 1.30, 3.94) increased the risk of DPN (**Supplementary Table 4**). After 1:1 matching with T2DM diagnosis date, sex, age, comorbidity index, utilization of medical resources, drug combination and other hypoglycemic agents (patient characteristics before and after matching are shown in **Supplementary Table 5**), the results were still consistent with the main results (**Supplementary Table 6**).

**Table 3:** Hazard ratios of diabetic peripheral neuropathy associated with metformin use according to subgroups.

Subgroups		Case	Total	Non-metformin (Ref)	Metformin	P-int*
Age, y	<60	1,065	26,367	1.00	2.29 (1.85, 2.84)	0.005
	≥60	868	23,338	1.00	1.57 (1.33, 1.84)	
Sex	Male	1,107	28,647	1.00	1.84 (1.56, 2.17)	0.996
	Female	826	21,058	1.00	1.85 (1.51, 2.28)	
CI-index	0	665	17,340	1.00	1.94 (1.56, 2.42)	0.815
	1	944	23,012	1.00	1.80 (1.49, 2.18)	
	≥2	324	9,353	1.00	1.74 (1.30, 2.33)	
Number of visits/y	0	820	20,827	1.00	2.39 (1.92, 2.98)	0.013
	1-2	392	9,342	1.00	1.48 (1.13, 1.93)	
	>2	721	19,536	1.00	1.62 (1.32, 1.97)	
Vitamin B12	Not use	1,892	48,451	1.00	1.84 (1.62, 2.10)	0.820
	Use	41	1,254	1.00	1.92 (0.79, 4.69)	
Vitamin B1	Not use	1,202	32,789	1.00	2.02 (1.70, 2.39)	0.068
	Use	731	16,916	1.00	1.62 (1.33, 1.97)	
Antihypertensive	Not use	552	13,225	1.00	2.11 (1.62, 2.75)	0.232
	Use	1,381	36,480	1.00	1.75 (1.51, 2.02)	
Antihyperlipidemic	Not use	619	16,677	1.00	2.17 (1.74, 2.71)	0.090
	Use	1,314	33,028	1.00	1.68 (1.44, 1.97)	
NSAIDs	Not use	999	27,219	1.00	2.11 (1.75, 2.55)	0.058
	Use	934	22,486	1.00	1.60 (1.34, 1.91)	

*P-value for interaction tests.

## Discussion

The current study found that metformin therapy increased the risk of hospital admission for peripheral neuropathy after diagnosis of T2DM. This increased risk was more likely to be observed after a long period of time and was not easily detected in a short period of time. A dose−response relationship was revealed between the daily dose of metformin and the increased risk of DPN, with high-dose metformin treatment (>2.0 g/d) associated with the highest risk of peripheral neuropathy.

Numerous studies have reported an association between metformin use and vitamin B12 deficiency and have raised the hypothesis of a potential peripheral neuropathy threat (5). A latest meta-analysis that included 31 studies showed that metformin use led to significantly lowered vitamin B12 concentrations and significantly higher risk of vitamin B12 deficiency in diabetic patients (6). Although plasma vitamin B12 levels have been widely found to be inversely associated with the risk of neuropathy (17, 18), several previous studies that explored the direct association between metformin and neuropathy did not find a significant association (6–10). However, these studies are few in number and poor in quality, such as small sample size, short follow-up, and lack of dose subgroups, so more high-quality studies are still needed to provide evidence. Our study is consistent with two previous cohort studies that have found an increased risk of DPN with long-term or high-dose metformin treatment. A retrospective cohort study using national Veterans Affairs data found that veterans over 50 years of age treated with metformin for at least 18 months were approximately 2-3 times more likely to develop DPN compared with those treated for at least 6 months but <18 months (11). Another clinical prospective study also found that metformin use within the previous 6 months of T2DM was associated with more severe DPN, especially in the high-dose group (12).

In the early phase after metformin administration, the good hypoglycemic effect of metformin may inhibit the progression of diabetic complications to a certain extent. Overall, however, our findings confirm the speculation of other previous studies that long-term metformin use can result in serum vitamin B12 deficiency, leading to exacerbating evidence of peripheral nerve damage. Furthermore, we found a positive association between metformin dose and DPN risk, possibly due to the fact that higher doses of metformin lead to more severe vitamin B12 deficiency. Previous studies have found that a 1 mg increase in daily metformin dose was associated with 0.042 (95% CI -0.060, -0.023) decrease in vitamin B12 concentrations (19). The mechanism by which metformin elevates the risk of DPN remains unclear to date (4, 20). One possible explanation is that metformin blocks the absorption of vitamin B12 from the gastrointestinal tract by interfering with the calcium-dependent binding of the intrinsic factor vitamin B12 complex to the cuboid receptor at the end of the ileum (21). Vitamin B12 is involved in the conversion of methylmalonyl-CoA and homocysteine, and higher concentrations of methylmalonyl-CoA and homocysteine have deleterious effects on the macrovascular system, including hematologic and neurological manifestations (11, 22).

In addition, we found that the adverse effect of metformin on DPN risk was more severe in T2DM patients with good glycemic control at baseline. That is, metformin treatment was associated with a higher risk of DPN in patients younger than 60 years of age, with fewer comorbidities, with lower utilization of medical resources, and not taking antihypertensive or lipid-lowering medications at baseline. In patients with poor baseline conditions (older age, more severe comorbidities, and more concomitant medications), the increased risk of DPN may be mainly due to lower glycosylated hemoglobin (hBA1c) levels as a result of more difficult glycemic control (23), and metformin treatment may contribute to some extent to glycemic control. These findings underscore the importance of adjusting medication prescriptions early in the course of diabetes to prevent peripheral neuropathy in patients with mild symptoms and low risk. We also found that metformin treatment did not increase the risk of DPN in patients with vitamin B12 prescription records at baseline, further suggesting the possible positive effect of early vitamin B12 supplementation in preventing DPN progression. Previous interventional studies have also demonstrated that B-vitamins may improve the symptoms of DPN (18). However, this finding needs to be interpreted with caution due to the small sample size of patients documented to be treated with vitamin B12 in this study.

This is the first study to examine the direct association between metformin treatment and the risk of peripheral neuropathy in Asian patients with T2DM. Our findings highlight the great benefits of early surveillance and adjustment of hypoglycemic agents for the prevention of peripheral neuropathy in patients with T2DM, especially in young patients with few comorbidities. In this study, all employees with T2DM in Beijing were followed for a long period of time to explore medication use and subsequent complication through a prospective cohort study. The subjects included in this study were all newly diagnosed T2DM patients, and the impact of the duration of T2DM on outcomes was controlled for as much as possible. Furthermore, we accounted for metformin use and average daily dose throughout the follow-up period, further reducing the possibility of misclassification due to changes in metformin prescription during follow-up. We also found that long-term and high-dose use of metformin caused more severe damage to peripheral nerves and provided recommendations for clinical treatment. In addition, the association between metformin treatment and DPN was assessed by controlling for multiple confounders and multiple sensitivity analyses. Finally, subgroup analyses were conducted to provide evidence to support precise prevention and treatment of patients with different characteristics.

This study has some limitations. First, the DPN cases identified in this study were all hospitalized cases, while most of the DPN cases were asymptomatic and undetected in the early stage. Therefore, our study cannot speculate on the effect of metformin treatment on all DPN cases. We can explain those with symptoms requiring hospitalization, who are more likely to have adverse outcomes also more in need of protection. Second, although the BMCDE database includes information on all the drugs prescribed by patients, inconsistencies between the drugs prescribed and the drugs actually taken by patients cannot be avoided. Patients with metformin prescriptions may not take metformin at all or in lower amounts, but those without a prescription almost certainly did not take metformin, skewing the results toward the null hypothesis and proving that our results are relatively conservative. Third, the timing of metformin initiation varied among patients, but our results showed that 69.6% patients started metformin within 6 months after diagnosis of T2DM. We excluded patients who developed DPN within 6 months and found that the risk was at least 60% higher in the treated group than in the nontreated group, regardless of when metformin therapy was initiated. Fourth, the metformin treatment group was likely to have more serious conditions at baseline than the non-metformin group. Therefore, to align disease severity at baseline between the exposed and non-exposed groups, patients with good adherence to insulin (considered to have poor glycemic control) or who had not taken any hypoglycemic agents within 6 months (considered to have mild symptoms) were further excluded from the sensitivity analyses. In addition, propensity scores were used to match baseline characteristics and comorbidities between the treated and nontreated groups. Fifth, concomitant use of other types of hypoglycemic drugs may have affected the interpretation of the results attributed to metformin. Thus, we also controlled for the use of other hypoglycemic drugs by enrolling patients using only one type of hypoglycemic drug and by matching them with the propensity score. Sixth, serum vitamin B12 levels, hBA1c and other biochemical markers that affect DPN were not available within the medical claims database, so we cannot accurately explain the association between metformin and DPN. Seventh, although we adjusted for confounding factors in as much detail as possible, risk factors (23) such as financial status and lifestyle, which were again unavailable within the database, still cannot be included in the model. Last, since the study was based on a homogenous Asian population, these results may be limited in extrapolation to other racial groups.

In conclusion, our results demonstrate that metformin treatment was associated with an increased long-term risk of hospitalization for peripheral neuropathy in Chinese patients with T2DM, and the risk was positively dose-responsive to the daily dose of metformin. In particular, metformin use was a major risk factor for DPN in younger patients. Concomitant vitamin B12 use may avoid the increased DPN risk associated with metformin use.

## Data availability statement

The data analyzed in this study is subject to the following licenses/restrictions: Restrictions apply to the availability of these data. Data were obtained from the administrative department of China’s health and medical system and are available with the permission of the administrative department. Requests to access these datasets should be directed to http://ybj.beijing.gov.cn/#.

## Ethics statement

The data were collected for an administrative purpose without any personal identifiers; therefore, Ethical review and approval was not required for the study on human participants in accordance with the local legislation and institutional requirements. Written informed consent for participation was not required for this study in accordance with the national legislation and the institutional requirements.

## Author contributions

RY, YH, and YW conceived and designed the paper. YH and YW designed and supervised the conduct of the whole study, obtained funding, and acquired the data. RY analyzed the data and drafted the manuscript. HY, JW, HC, MW, SW, XQ and TW contributed to the interpretation of the results and critical revision of the manuscript for important intellectual content. All authors approved the final version of the manuscript.
