# Supplementary material for: Metformin treatment and risk of diabetic peripheral neuropathy in patients with type 2 diabetes mellitus in Beijing, China
Source: Front Endocrinol (Lausanne). 2023 Feb 28;14:1082720. doi: 10.3389/fendo.2023.1082720 (PMC10011647; doi:10.3389/fendo.2023.1082720)
Supplement: Supplementary file 1 [file Table_1.docx]

**Supplementary tables**

**Supplementary table 1.** Hazard ratios of diabetic peripheral neuropathy associated with metformin use and average daily dose of metformin use, excluding patients who had no record of any type of hypoglycemic drug prescription within 6 months.

|  | Case | Total | Cases/PYs (/1000) | HR |
| --- | --- | --- | --- | --- |
| Non-metformin | 279 | 11,647 | 4.23 | 1.00 |
| Metformin | 1,630 | 36,213 | 7.20 | 1.72 (1.51, 1.96) |
| Daily dose, g |  |  |  |  |
| <1.0 | 461 | 18,978 | 3.83 | 0.92 (0.79, 1.07) |
| 1.0-2.0 | 295 | 8,064 | 5.80 | 1.42 (1.21, 1.68) |
| >2.0 | 874 | 9,171 | 15.82 | 3.94 (3.44, 4.53) |

**Supplementary table 2.** Hazard ratios of diabetic peripheral neuropathy associated with metformin use and average daily dose of metformin use, excluding patients who had used insulin within 6 months.

|  | Case | Total | Cases/PYs (/1000) | HR |
| --- | --- | --- | --- | --- |
| Non-metformin | 51 | 3,089 | 2.75 | 1.00 |
| Metformin | 342 | 9,929 | 5.45 | 1.88 (1.40, 2.54) |
| Daily dose, g |  |  |  |  |
| <1.0 | 31 | 1,642 | 2.98 | 0.87 (0.61, 1.25) |
| 1.0-2.0 | 139 | 4,195 | 5.27 | 1.43 (0.98, 2.08) |
| >2.0 | 171 | 1,067 | 27.86 | 4.67 (3.41, 6.40) |

**Supplementary table 3.** Hazard ratios of diabetic peripheral neuropathy associated with metformin use and average daily dose of metformin use, excluding patients who developed DPN within 12 months to observe the long-term effects of metformin.

|  | Case | Total | Cases/PYs (/1000) | HR |
| --- | --- | --- | --- | --- |
| Non-metformin | 246 | 12,277 | 3.42 | 1.00 |
| Metformin | 1576 | 36,839 | 6.81 | 2.00 (1.74, 2.29) |
| Daily dose, g |  |  |  |  |
| <1.0 | 441 | 19,562 | 3.54 | 1.04 (0.89, 1.22) |
| 1.0-2.0 | 286 | 8,162 | 5.54 | 1.67 (1.40, 1.98) |
| >2.0 | 849 | 9,115 | 15.33 | 4.71 (4.07, 5.44) |

**Supplementary table 4.** Hazard ratios of diabetic peripheral neuropathy associated with metformin use and average daily dose of metformin use, retaining patients who used only one type of hypoglycemic drugs.

|  | Case | Total | Cases/PYs (/1000) | HR |
| --- | --- | --- | --- | --- |
| Non-metformin | 169 | 4,762 | 6.32 | 1.00 |
| Metformin | 32 | 464 | 11.29 | 1.62 (1.10, 2.39) |
| Type of hypoglycemic drugs | | |  |  |
| Glycosidase inhibitors | 22 | 841 | 4.43 | 1.00 |
| Metformin | 32 | 464 | 11.29 | 2.27 (1.30, 3.94) |
| Thiazolidinediones | 1 | 27 | 6.03 | 1.26 (0.17, 9.37) |
| Glinides | 5 | 141 | 5.83 | 1.24 (0.47, 3.29) |
| Sulfonylureas | 6 | 186 | 5.62 | 1.27 (0.51, 3.14) |
| Insulin | 135 | 3567 | 6.86 | 1.51 (0.96, 2.38) |

**Supplementary table 5.** Baseline characteristics of study participants, before and after matching metformin-treated and non-metformin-treated groups 1:1 by logit propensity score (for sex, age, date of diagnosis of T2DM, comorbidities index, number of visits, concomitant medication, and other hypoglycemic agents).

| Variable |  | Non-metformin | Metformin | SMD*, % | P-value |
| --- | --- | --- | --- | --- | --- |
| Date of diagnosis of T2DM | Unmatched | 2011/5/25 | 2011/5/25 | -1.8 | 0.074 |
|  | Matched | 2011/5/28 | 2011/5/28 | -0.1 | 0.922 |
| Age, y | Unmatched | 63.93 | 58.16 | -46.6 | <0.001 |
|  | Matched | 58.17 | 64.02 | -44.7 | <0.001 |
| Female, % | Unmatched | 63.89 | 63.45 | 2.7 | 0.007 |
|  | Matched | 0.43 | 0.39 | 8.2 | <0.001 |
| Comorbidity index | Unmatched | 0.39 | 0.40 | -1.5 | 0.126 |
|  | Matched | 0.90 | 0.95 | -6.3 | <0.001 |
| Number of visits/y | Unmatched | 0.95 | 0.95 | 0.1 | 0.955 |
|  | Matched | 13.96 | 15.03 | -4.1 | <0.001 |
| Vitamin B12, % | Unmatched | 14.90 | 15.01 | -0.3 | 0.736 |
|  | Matched | 0.02 | 0.03 | -2.0 | 0.051 |
| Vitamin B1, % | Unmatched | 0.03 | 0.03 | -0.3 | 0.753 |
|  | Matched | 0.34 | 0.35 | -3.8 | <0.001 |
| Antihypertensive, % | Unmatched | 0.35 | 0.35 | 0.1 | 0.936 |
|  | Matched | 0.72 | 0.77 | -10.4 | <0.001 |
| Antihyperlipidemic, % | Unmatched | 0.77 | 0.77 | 0.3 | 0.774 |
|  | Matched | 0.68 | 0.61 | 14.2 | <0.001 |
| NSAIDs, % | Unmatched | 0.61 | 0.63 | -2.3 | 0.021 |
|  | Matched | 0.45 | 0.47 | -4.1 | <0.001 |
| Glinides, % | Unmatched | 0.19 | 0.12 | 17.1 | <0.001 |
|  | Matched | 0.11 | 0.12 | -2.6 | 0.011 |
| Sulfonylureas, % | Unmatched | 0.32 | 0.19 | 27.7 | <0.001 |
|  | Matched | 0.18 | 0.19 | -2.5 | 0.011 |
| Thiazolidinediones, % | Unmatched | 0.10 | 0.04 | 21.6 | <0.001 |
|  | Matched | 0.03 | 0.04 | -2.4 | 0.019 |
| Glycosidase inhibitors, % | Unmatched | 0.61 | 0.56 | 9.6 | <0.001 |
|  | Matched | 0.56 | 0.57 | -0.5 | 0.642 |
| Insulin, % | Unmatched | 0.73 | 0.76 | -5.3 | <0.001 |
|  | Matched | 0.76 | 0.75 | 0.3 | 0.789 |

*SMD, standardized mean difference. SMD of acceptable matching is less than 0.1.

**Supplementary table 6.** Hazard ratios of diabetic peripheral neuropathy associated with metformin use and average daily dose of metformin use after matching metformin-treated and non-metformin-treated groups 1:1 by logit propensity score (for sex, age, date of diagnosis of T2DM, comorbidities index, number of visits, concomitant medication, and other hypoglycemic agents).

|  | Case | Total | Cases/PYs (/1000) | HR |
| --- | --- | --- | --- | --- |
| Non-metformin | 274 | 12,236 | 3.90 | 1.00 |
| Metformin | 530 | 12,236 | 7.09 | 1.81 (1.56, 2.09) |
| Daily dose, g | (Continuous) |  |  | 1.42 (1.38, 1.46) |
| <1.0 | 180 | 7,028 | 4.13 | 1.03 (0.85, 1.25) |
| 1.0-2.0 | 103 | 2,481 | 6.75 | 1.73 (1.38, 2.17) |
| >2.0 | 247 | 2,727 | 15.49 | 3.98 (3.35, 4.73) |
